# Progressive myoclonus epilepsy *KCNC1* variant causes a developmental dendritopathy

**DOI:** 10.1111/epi.16867

**Published:** 2021-03-18

**Authors:** Jenna C. Carpenter, Roope Männikkö, Catherine Heffner, Jana Heneine, Marisol Sampedro‐Castañeda, Gabriele Lignani, Stephanie Schorge

**Affiliations:** ^1^ Department of Clinical and Experimental Epilepsy University College London Queen Square Institute of Neurology London UK; ^2^ Department of Neuromuscular Diseases University College London Queen Square Institute of Neurology London UK; ^3^ Department of Pharmacology University College London School of Pharmacy London UK

**Keywords:** dendrites, dendritopathy, development, *KCNC1*, K_V_3.1, MEAK, potassium channels, progressive myoclonic epilepsy

## Abstract

**Objective:**

Mutations in *KCNC1* can cause severe neurological dysfunction, including intellectual disability, epilepsy, and ataxia. The Arg320His variant, which occurs in the voltage‐sensing domain of the channel, causes a highly penetrant and specific form of progressive myoclonus epilepsy with severe ataxia, designated myoclonus epilepsy and ataxia due to potassium channel mutation (MEAK). *KCNC1* encodes the voltage‐gated potassium channel K_V_3.1, a channel that is important for enabling high‐frequency firing in interneurons, raising the possibility that MEAK is associated with reduced interneuronal function.

**Methods:**

To determine how this variant triggers MEAK, we expressed K_V_3.1b^R320H^ in cortical interneurons in vitro and investigated the effects on neuronal function and morphology. We also performed electrophysiological recordings of oocytes expressing K_V_3.1b to determine whether the mutation introduces gating pore currents.

**Results:**

Expression of the K_V_3.1b^R320H^ variant profoundly reduced excitability of mature cortical interneurons, and cells expressing these channels were unable to support high‐frequency firing. The mutant channel also had an unexpected effect on morphology, severely impairing neurite development and interneuron viability, an effect that could not be rescued by blocking K_V_3 channels. Oocyte recordings confirmed that in the adult K_V_3.1b isoform, R320H confers a dominant negative loss‐of‐function effect by slowing channel activation, but does not introduce potentially toxic gating pore currents.

**Significance:**

Overall, our data suggest that, in addition to the regulation of high‐frequency firing, K_V_3.1 channels play a hitherto unrecognized role in neuronal development. MEAK may be described as a developmental dendritopathy.

**Key Points**

- We expressed the PME/MEAK variant, K_V_3.1b^R320H^, in neurons
- Mature neurons expressing K_V_3.1b^R320H^ cannot sustain high‐frequency firing
- K_V_3.1b^R320H^ alters neurodevelopment
- K_V_3.1b^R320H^ reduces dendritic length and arborization
- PME/MEAK might represent a developmental dendritopathy

## 1 INTRODUCTION

Progressive myoclonus epilepsy (PME) encompasses a clinically heterogeneous group of severe monogenic disorders, characterized by the core symptoms of myoclonus, epilepsy, and neurological deterioration, often in the form of ataxia and/or dementia.^1^, ^2^ PME is drug‐resistant and relentlessly progressive, often resulting in reduced life expectancy, severe motor dysfunction, and cognitive decline.^3^ Insights into PME disease mechanisms are urgently required to guide the rational development of new therapeutics.

A series of rare variants cause PME. Of these, a dominantly inherited de novo missense mutation in *KCNC1* (c.959 G > A, p.R320H), which encodes the voltage‐gated potassium channel (VGKC) K_V_3.1, causes PME with ataxia (myoclonus epilepsy and ataxia due to potassium channel mutation [MEAK]; Online Mendelian Inheritance in Man database [OMIM] # 616187).^4^, ^5^ K_V_3.1 belongs to the K_V_3 family of high‐voltage activated VGKCs (K_V_3.1–K_V_3.4, encoded by *KCNC1*–*KCNC4*)^6^ and is predominantly expressed in cortical fast‐spiking parvalbumin‐positive interneurons, which are fundamental in providing feedforward and feedback inhibition to regulate network oscillations, and neurons of the cerebellum in both rodents and humans.^7^, ^8^ The fast kinetics of the channel enable firing at frequencies beyond 200 Hz via the rapid repolarization of the action potential (AP; reviewed in Kaczmarek and Zhang,^7^, ^9^, ^10^).

K_V_3.1 undergoes alternative splicing to generate two channel isoforms, K_V_3.1a and K_V_3.1b.^7^ In the adult brain, K_V_3.1b is the dominant splice isoform, reaching peak expression around adolescence, roughly coinciding with symptom onset in MEAK.^5^, ^11^ K_V_3.1a predominates during early embryonic development.^12^, ^13^ K_V_3.1b has a longer C‐terminus that forms a distinct site for the posttranslational regulation of channel function by phosphorylation.^14^ In vivo, K_V_3.1a is mainly localized to the axon, whereas K_V_3.1b is more broadly distributed, including in the proximal dendrites.^15^, ^16^, ^17^

The R320H mutation in K_V_3.1 removes the highly conserved fourth arginine of the S4 voltage sensor of the channel and produces a dominant negative loss‐of‐function in both splice variants.^4^, ^5^, ^18^ However, the specific effects of R320H are dependent on splice variant and heterologous expression system.^4^, ^5^, ^18^ Mutations removing voltage‐sensing arginines can introduce pathogenic gating pore currents through the voltage sensor domain (VSD).^19^, ^20^ In the *Drosophila* K_V_1 (Shaker), the analogous arginine to histidine mutation of the fourth voltage sensing arginine (K_V_1.1^R371H^) introduces a proton‐selective pore in the VSD that opens upon depolarization.^19^ These gating pore currents constitute the pathogenic mechanism for some muscle channelopathies,^21^, ^22^ but have not yet convincingly been associated with neuronal channelopathies. The conserved role of the S4 arginines raises the possibility that a proton gating pore current through K_V_3.1^R320H^ contributes to pathology in MEAK.

Other loss‐of‐function variants in *KCNC1* cause human neurological diseases distinct from PME. A nonsense variant (c.1015 C > T, p.R339X) was identified in three affected individuals of a single family with intellectual disability without seizures.^23^ A recurrent A421V variant is associated with developmental and epileptic encephalopathy (DEE).^24^, ^25^ *KCNC1* variants have also been associated with developmental encephalopathy without seizures^24^ and intellectual disability.^24^, ^25^ The phenotypic spectrum for *KCNC1* variants suggests that they have variable effects during development, potentially involving different disease mechanisms.^7^, ^26^

Here, we show that when expressed in neurons in vitro, channels containing the MEAK variant, K_V_3.1^R320H^, reduce interneuronal excitability and high‐frequency firing, as predicted for a dominant loss‐of‐function variant. Unexpectedly, mutant channel expression in interneurons also produces a profound impairment in dendritic growth. K_V_3.1b^R320H^ does not conduct gating‐pore currents, indicating instead that the slowed kinetics of channel gating underlie MEAK. Overall, we suggest that MEAK may represent a developmental dendritopathy.

## 2 MATERIALS AND METHODS

### 2.1 Animals and ethics

Animal care and experimental procedures were carried out in accordance with the UK Animals (Scientific Procedures) Act 1986.

### 2.2 Molecular biology

The c.959 G > A PME mutation, which results in the substitution of arginine 320 with histidine, was introduced into the human *KCNC1* coding sequence for the b isoform (accession # NM_001112741.2). K_V_3.1b expression was restricted to interneurons using the mouse Dlx5/6 (mDlx5/6) enhancer/promoter.^27^

The R371H gating pore mutation was introduced into the nonconducting (W434F),^28^ fast‐inactivation removed (Δ6‐46)^29^ Shaker H4 K^+^ channel (accession # NM_167595.3).

### 2.3 Channel expression in *Xenopus* oocytes

Oocytes were injected with a total of 2.5 ng of complementary RNA (cRNA). When studying K_V_3.1 heteromers, K_V_3.1b^WT^ and K_V_3.1b^R320H^ cRNAs were coinjected at a 1:1 mass ratio for a total of 2.5 ng of cRNA.

### 2.4 Electrophysiological recordings of oocytes

Two‐electrode voltage clamp oocyte recordings were obtained 48–72 h after oocyte injection. For all recordings, electrodes were filled with 3 mol·L^–1^ KCl solution (pH 7.4) and had a tip resistance of .1–.5 MΩ. The bath solution contained 120 mmol·L^–1^ Na^+^‐methanesulfonate (NaMeSO_4_)_, _120 mmol·L^–1^ CH_3_SO_3_Na, 1.8 mmol·L^–1^ CaSO_4_, and 10 mmol·L^–1^ hydroxyethylpiperazine ethane sulfonic acid (HEPES) at pH 7.4. For gating pore current recordings, oocytes were perfused first with NaMeSO_4 _solution at pH 7.4 followed by NaMeSO_4_ at pH 5.5.

Oocytes were held at a potential of −80 mV. The voltage protocol used to measure whole cell K^+^ currents consisted of 250‐ms test voltage steps ranging from −100 mV to +60 mV (Δ10 mV), followed by a tail step to −30 mV for 250‐ms, carried out in the presence of the ‐*P*/4 leak subtraction protocol. Gating pore current recordings were carried out in the absence of leak subtraction protocols, using the same voltage protocol described above.

### 2.5 Lentiviral production

Vesicular stomatitis virus glycoprotein pseudotyped second‐generation human immunodeficiency virus type 1‐based lentiviral particles were generated using previously described methods. Functional lentiviral titers were calculated using genomic DNA extracted from transduced human embryonic kidney (HEK) cells.

### 2.6 Primary neuronal culture

Primary cortical neuronal cultures were prepared from postnatal day 0–1 C57BL/6 J mouse pup cortices according to a previously described protocol. Cortical cultures were transfected at 4–5 days in vitro (DIV) using magnetofection (OZ Biosciences). When assessing the effect of K_V_3 blockade on dendritic growth, media were supplemented with tetraethylammonium chloride (1 mmol·L^–1^ in water, Sigma) or 100 mmol·L^–1^ iberiotoxin (IbTX). Lentiviral transduction of cultures was carried out at 1–2 DIV.

### 2.7 Immunocytochemistry

Cortical neurons were fixed in 4% paraformaldehyde in phosphate‐buffered saline. Images were acquired using an inverted LSM 710 (Zeiss) confocal laser scanning microscope (ZEN software, 2009) with ×20 objective or ×40 or ×63 EC Plan‐Neofluar oil‐immersion objective (Zeiss). The following primary antibodies were used: rabbit α‐K_V_3.1b (1:500; Alomone Labs), SMI‐31 (1:1000; BioLegend), guinea pig α‐microtubule associated protein 2 (α‐MAP2; 1:1000; Synaptic Systems).

### 2.8 Image analysis

Image analysis was performed using ImageJ (v1.51u). Neurites were semiautomatically traced using the NeuronJ plugin.^30^ Sholl analysis was performed on traced dendrites using a step size of 1 μm.

### 2.9 Electrophysiological recordings of neuronal cultures

Whole cell current‐clamp electrophysiological recordings of cortical neurons were carried out at 14–16 DIV. Cells were held at −70 mV in current clamp configuration. Recordings were carried out at 32 °C with continuous oxygenated perfusion. The internal solution contained (in mmol·L^–1^) 148 K‐gluconate, 4 NaCl, 1 MgSO_4_, .02 CaCl_2_, .1 1,2‐bis(o‐aminophenoxy)ethane‐N,N,N′,N′‐tetraacetic acid, 15 glucose, 5 HEPES, 3 adenosine triphosphate, and .1 guanosine triphosphate. The external solution contained (in mmol·L^–1^) 119 NaCl, 25 NaHCO_3_, 11 glucose, 2.5 KCl, 1.25 NaH_2_PO_4_, 2.5 CaCl_2_, and 1.3 MgCl_2_. A current step protocol was used to trigger APs by injecting currents ranging from –20 pA to +300 pA (Δ10 pA) for 1‐s. To investigate cellular firing frequencies (from 10 to 100 Hz), neurons were injected with 10 current pulses at 110% of the AP current threshold using a 5‐ms stimulus. The current threshold was found by iteratively injecting neurons with 5‐ms depolarizing steps (Δ10 pA) until an AP was fired.

### 2.10 Statistics

Data are plotted as scatter plots, representing single data points. Box plots show the mean (+), median (middle line), percentiles (25%–75%) and maximum/minimum points where represented. One‐way analysis of variance (ANOVA) was used to compare three groups and was followed by a post hoc test for functional analysis. To compare two groups at different time points, a two‐way repeated measure ANOVA, followed by a post hoc test for functional analysis, was used. Statistical analysis was carried out using Prism with significance set at *p* < .05.

## 3 RESULTS

### 3.1 K_V_3.1b^R320H^ reduces the excitability of mature interneurons

**FIGURE 1:**
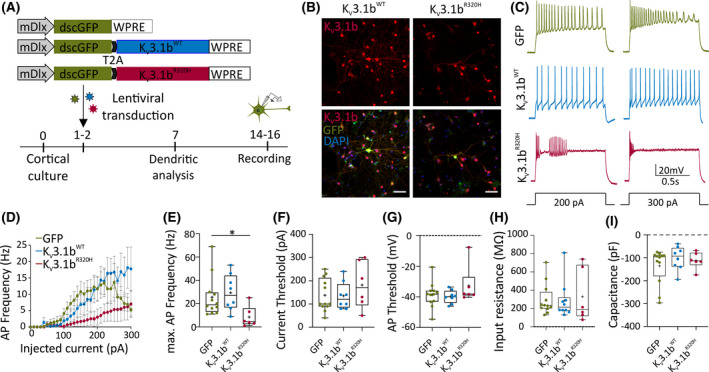
K_V_3.1b^R320H^ reduces interneuronal excitability. (A) Experimental scheme. (B) Representative immunofluorescent images of neurons at 14 days in vitro expressing K_V_3.1b channel variants. (C) Representative traces of interneuronal firing with 1‐s, 200 or 300 pA current injections. (D) Firing frequencies with incremental current injections of interneurons expressing K_V_3.1b^WT^, K_V_3.1b^R320H^, or green fluorescent protein (GFP) only (*p* = .016; repeated measures two‐way analysis of variance [ANOVA] followed by Bonferroni multiple comparison test). (E) Maximum firing frequency (K_V_3.1b^R320H^ vs. GFP: *p = .043; K_V_3.1b^WT^ vs. GFP: *p* > .99, Kruskal–Wallis test followed by Dunn multiple comparisons test). (F) Minimal current amplitude required to elicit an action potential (AP; K_V_3.1b^R320H^ vs. GFP: *p* = .50; K_V_3.1b^WT^ vs. GFP: *p* > .99). (G–I) There were no significant differences in AP threshold (G; K_V_3.1b^R320H^ vs. GFP: *p* = .34; K_V_3.1b^WT^ vs. GFP: *p* > .99), input resistance (H; K_V_3.1b^R320H^ vs. GFP: *p* > .99; K_V_3.1b^WT^ vs. GFP: *p* > .99), or capacitance (I; K_V_3.1b^R320H^ vs. GFP: *p* = .91; K_V_3.1b^WT^ vs. GFP: *p* = .51). All comparisons in F–I were performed with one‐way ANOVA with Bonferroni multiple comparison test. DAPI, 4′,6‐diamidino‐2‐phenylindole (nuclear stain); mDlx, mouse Dlx; WT, wild type; dscGFP: destabilized copGFP; WPRE: Woodchuck Hepatitis Virus Posttranscriptional Regulatory Element

Because K_V_3.1 plays a major role in supporting high‐frequency firing, we first asked whether the expression of K_V_3.1b^R320H^ is sufficient to disrupt neuronal firing, particularly during high‐frequency trains. To model the dominant effects of the R320H mutation on K_V_3 function, we used lentiviruses to overexpress K_V_3.1b variants (K_V_3.1^WT^ and K_V_3.1^R320H^) and green fluorescent protein (GFP) in cortical neuronal cultures. Transgene expression was restricted to interneurons using the mDlx5/6 promoter, which was found to be highly specific (Figure S1; 93% ± 1.9% of transduced neurons were positive for glutamate decarboxylase 67), and electrophysiological recordings were performed from mature transduced interneurons at 14–16 DIV (Figure 1A). Both K_V_3.1^WT^‐expressing and K_V_3.1^R320H^‐expressing neurons had significantly different input–output relationships from GFP controls. As expected, expression of K_V_3.1b^WT^ enabled high‐frequency firing and prevented depolarization block in response to current injections greater than 200 pA (Figure 1C–D). In contrast, K_V_3.1b^R320H^ significantly reduced AP frequency compared to GFP across a range of current steps (Figure 1C–E). There were no significant differences in the current threshold, AP threshold, input resistance, or capacitance (Figure 1F–I).

### 3.2 K_V_3.1b^R320H^ does not support high‐frequency firing in interneurons

**FIGURE 2:**
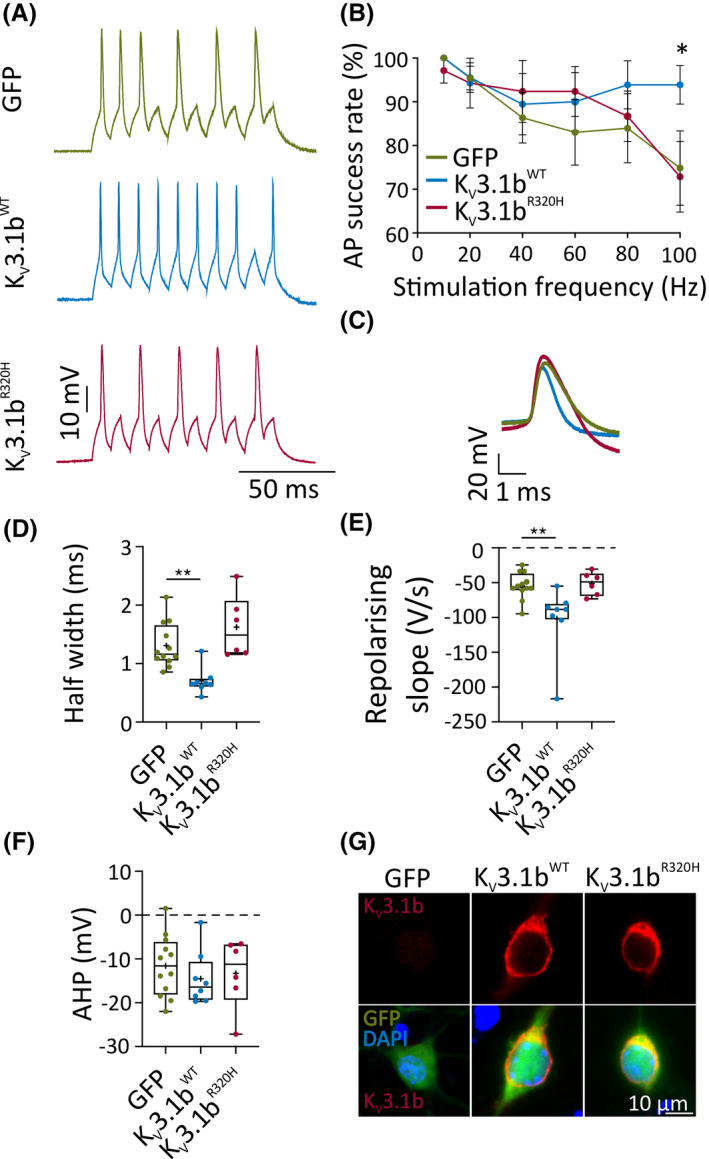
K_V_3.1b^R320H^ cannot sustain high‐frequency interneuron firing. (A) Representative traces of interneuronal firing at 14–16 days in vitro (DIV) expressing green fluorescent protein (GFP) only, K_V_3.1b^WT^, or K_V_3.1b^R320H^ (100 Hz, 5 ‐ms). (B) Action potential (AP) success rate when stimulated at different frequencies (100 Hz: K_V_3.1b^WT^ vs. GFP: *p = .04, two‐way repeated measures analysis of variance [ANOVA] followed by Bonferroni multiple comparison test). (C) Representative AP waveforms of interneurons at 14–16 DIV expressing GFP only or K_V_3.1b channel variants. (D, E) AP half‐width (**p = .004) and the rate of AP repolarization (**p = .005) of K_V_3.1b^WT^ neurons compared to GFP control neurons (one‐way ANOVA with Bonferroni multiple comparison test). (F) Afterhyperpolarization (AHP) of the first AP. (G) Representative confocal images of lentivirally transduced interneurons at 14 DIV, immunolabeled for K_V_3.1b. DAPI, 4′,6‐diamidino‐2‐phenylindole (nuclear stain); WT, wild type

To assess the impact of K_V_3.1b^R320H^ channels on high‐frequency firing in response to rapid current inputs, we delivered 5‐ms suprathreshold current pulses at frequencies of 10–100 Hz (Figure 2A). Neurons overexpressing K_V_3.1^WT^ had significantly higher AP success rates at 100 Hz compared to GFP control neurons (Figure 2B). Overexpression of K_V_3.1b^R320H^, on the other hand, had no effect on spike fidelity at 100 Hz compared to GFP controls (Figure 2B). The lack of effect of K_V_3.1b^R320H^ on firing in response to stimulus trains may be due to the repolarization after the end of the step being sufficient to support firing at this rate, or to low functional expression of endogenous K_V_3.1b at this developmental stage in culture (Figure 2G). The somatic expression level of K_V_3.1b was not found to differ for the wild‐type (WT) and mutant channel (Figure S2).

In these experiments, overexpression of K_V_3.1b^WT^ significantly increased the rate of repolarization and decreased AP half‐width compared to GFP (Figure 2C–E). In contrast, K_V_3.1b^R320H^ did not significantly affect these parameters (Figure 2C–E). K_V_3.1b variants had no effect on the afterhyperpolarization amplitude (Figure 2F), or on AP voltage threshold, rising slope, or amplitude (Table S1).

### 3.3 Overexpression of K_V_3.1b^R320H^ impairs dendritic development

**FIGURE 3:**
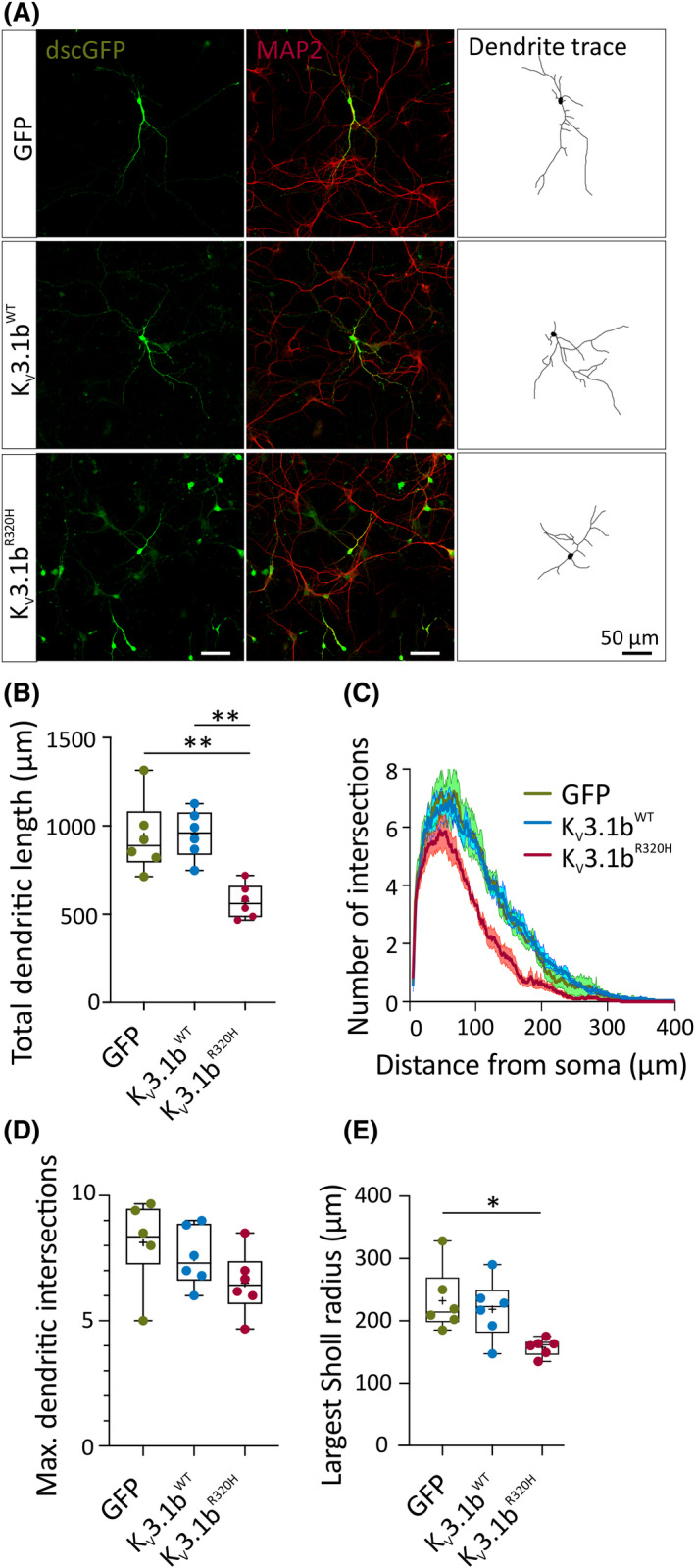
Lentiviral‐mediated overexpression of K_V_3.1b^R320H^ impairs interneuron dendritic development. (A) Representative confocal images at 7 days in vitro of neurons transduced with green fluorescent protein (GFP), K_V_3.1b^WT^, or K_V_3.1b^R320H^ lentiviruses. (B) Total dendritic length (GFP vs. K_V_3.1b^R320H^: **p = .003; K_V_3.1b^WT^ vs. K_V_3.1b^R320H^: **p = .002, GFP vs. K_V_3.1b^WT^: *p* > .99, one‐way analysis of variance [ANOVA] with Bonferroni multiple comparison test). (C) Sholl curves generated from the analysis of traced dendritic arbors. (D) Maximum number of dendritic intersections with Sholl analysis (GFP vs. K_V_3.1b^R320H^: *p* = .18; K_V_3.1b^WT^ vs. K_V_3.1b^R320H^: *p* = .64, GFP vs. K_V_3.1b^WT^: *p* > .99, one‐way ANOVA with Bonferroni multiple comparison test). (E) Maximum Sholl radius, indicating maximal dendritic length (GFP vs. K_V_3.1b^R320H^: *p = .021; one‐way ANOVA with Bonferroni multiple comparison test). *n* indicates the average of 5/6 neurons analyzed per coverslip. MAP2, microtubule‐associated protein 2 (dendritic marker); WT, wild type; dscGFP: destabilized copGFP.

Interneurons expressing K_V_3.1b^R320H^ had clear morphological defects at 14–16 DIV (Figure 1B). To quantify this, we transduced neurons at 1 DIV, when neurons undergo a rapid period of neuritogenesis, and performed dendritic analysis at 7 DIV (Figure 3A). A significantly higher percentage of neurons expressing K_V_3.1b^R320H^ had undetectable processes at 7 DIV (85.7% ± 1.1%, *n* = 6) compared to WT (27.8% ± 2.6%, *n* = 6) and GFP controls (30.9% ± 4.0%, *n* = 6; *p* < .001, one‐way ANOVA followed by Bonferroni multiple comparison test). Of those neurons with clearly detectable processes, K_V_3.1b^R320H^ expression had no significant effect on dendritic arborization, but significantly reduced total dendritic length when compared to GFP and K_V_3.1b^WT^ controls. In contrast, overexpression of K_V_3.1b^WT^ had no effect on either total dendritic length or dendritic arborization compared to GFP controls (Figure 3B–E). Immunolabeling neurons with the dendritic marker MAP2 confirmed transgenic expression of K_V_3.1b throughout the dendrites for both WT and mutant channels (Figure S2D).

### 3.4 Acute expression of K_V_3.1b^R320H^ impairs neurite development

**FIGURE 4:**
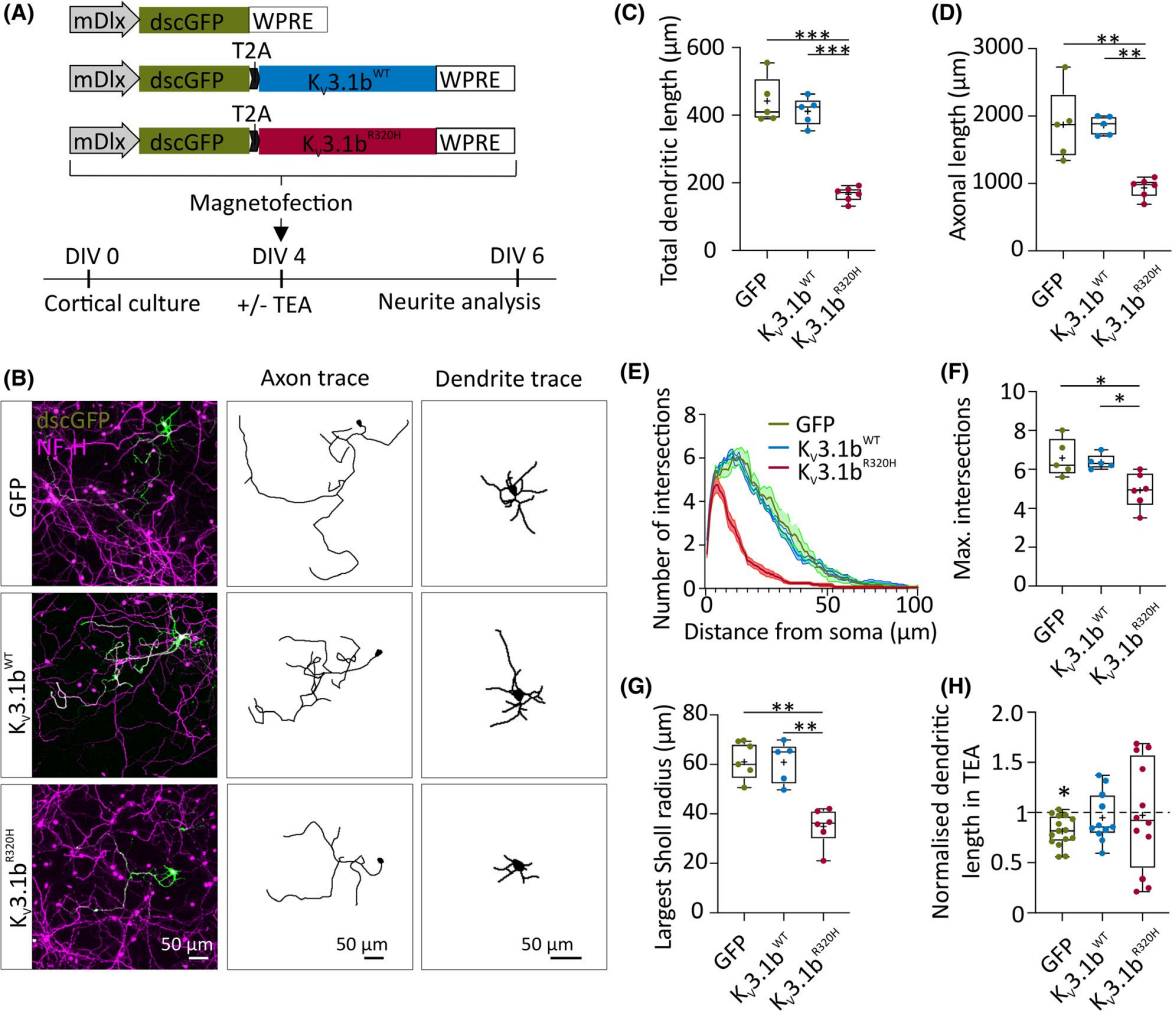
K_V_3.1b^R320H^ impairs neurite development. (A) Experimental scheme. (B) Representative epifluorescence images and dendritic and axonal tracings of neurons transfected with green fluorescent protein (GFP), K_V_3.1b^WT^, or K_V_3.1b^R320H^. (C) Total dendritic length 48 h after transfection (GFP vs. K_V_3.1b^WT^: *p* = .98; GFP vs. K_V_3.1b^R320H^: ***p < .001; K_V_3.1b^R320H^ vs. K_V_3.1b^WT^: ***p < .001, one‐way analysis of variance [ANOVA] with Bonferroni multiple comparison test). (D) Axonal length after 48 h of transduction (GFP vs. K_V_3.1b^WT^: *p* > .99; GFP vs. K_V_3.1b^R320H^: **p = .001; K_V_3.1b^WT^ vs. K_V_3.1b^R320H^: **p = .001, one‐way ANOVA with Bonferroni multiple comparison test). (E) Sholl analysis performed on traced dendritic arbors at 6 days in vitro (DIV). (F) Dendritic branching and the maximum number of dendritic intersections after 48 h of transduction (GFP vs. K_V_3.1b^WT^: *p* > .99; GFP vs. K_V_3.1b^R320H^: *p = .01; K_V_3.1b^R320H^ vs. K_V_3.1b^WT^:*p = .03, one‐way ANOVA with Bonferroni multiple comparison test). (G) Largest radius used in Sholl analysis after 48 h of transduction (GFP vs. K_V_3.1b^WT^: *p* > .99; GFP vs. K_V_3.1b^R320H^:***p < .001; K_V_3.1b^R320H^ vs. K_V_3.1b^WT^: ***p < .001, one‐way ANOVA with Bonferroni multiple comparison test). (H) Dendritic length after pharmacological block of K_V_3 channels with tetraethylammonium (TEA). Neurons were treated for 48 h with 1 mmol·L^–1^ TEA immediately after transfection (Student *t*‐test, treated vs. untreated: *p = .002; Student *t*‐test: K_V_3.1b^WT^, treated vs. untreated: *p* = .54; K_V_3.1b^R320H^, treated vs. untreated: *p* = .89; these data are corrected for multiple comparisons where *α* = .016). NF‐H, neurofilament heavy polypeptide (axonal marker). mDlx, mouse Dlx; NF‐H, neurofilament heavy polypeptide (axonal marker); WT, wild type; dscGFP: destabilized copGFP; WPRE: Woodchuck Hepatitis Virus Posttranscriptional Regulatory Element

To assess whether K_V_3.1b^R320H^ reduces dendritic outgrowth or causes dendritic collapse, we introduced K_V_3.1b variants by plasmid transfection at 4 DIV, a time point at which the dendritic arbor is partly established (Figure 4A). GFP signal was detected within 12 h, and neurons were analyzed at 24 and 48 h after transfection. At 48 h, K_V_3.1b^WT^ had no effect on dendritic length or arborization with respect to GFP controls, whereas K_V_3.1b^R320H^ expression significantly reduced dendritic length (Figure 4B–G). Likewise, K_V_3.1b^R320H^ significantly reduced axonal length, whereas K_V_3.1b^WT^ had no effect (Figure 4D). Unlike lentiviral overexpression, transfection of K_V_3.1b^R320H^ also resulted in a significant reduction in dendritic arborization, compared to GFP and K_V_3.1b^WT^ controls (Figure 4C,D; Figure S1). Potentially, this difference is caused by higher levels of channel expression after plasmid transfection (Figure 4E,F,G).

Dendritic length did not decrease between 24 and 48 h after transfection with K_V_3.1b^R320H^, suggesting that reduced length at 48 h is due to impaired outgrowth and not collapse (Figure S3). The axons of K_V_3.1b^R320H^ neurons also remained stable between 24 and 48 h (Figure S3), but unlike dendrites, axonal length at 24 h was still similar to controls, suggesting that dendritic growth was affected before axonal growth (Figure S3).

### 3.5 Overexpression of K_V_3.1b^R320H^ leads to cell death

In addition to compromised dendritic development, a higher proportion of neurons expressing K_V_3.1b^R320H^ were positive for a marker of programmed cell death (terminal deoxynucleotide transferase‐mediated deoxyuridine triphosphate nick‐end labeling [TUNEL]) after 72 h, with respect to GFP and K_V_3.1b^WT^ (Figure S5). At 72 h posttransfection, K_V_3.1b^R320H^ neurons had rounded cell bodies and blebbing of neuronal processes, consistent with programmed cell death. K_V_3.1b^R320H^ neurons were still viable 48 h after transfection as indicated by the TUNEL assay (Figure S5), but there were early proapoptotic nuclear changes, such as chromatin condensation and morphological irregularities, reflected in a significantly reduced nuclear area factor (Figure S6). At 24 h, there were no nuclear changes in K_V_3.1b^R320H^‐expressing neurons (Figure S6). These data suggest that the early developmental alterations induced by K_V_3.1b^R320H^ expression reduce neuronal viability.

### 3.6 Pharmacological block of K_V_3 channels does not prevent the adverse effects of K_V_3.1b^R320H^ on dendritic development

To examine whether altered kinetics of potassium currents through K_V_3.1b^R320H^ contribute to defects in neurite development, we treated cells immediately after transfection, before channel expression would be expected, with 1 mmol·L^–1^ tetraethylammonium (TEA), a concentration that blocks all K_V_3 family members and a subset of other K^+^ channels.^31^ Although TEA did impose a small but significant decrease in dendritic length in GFP controls (Figure 4H), it did not significantly change the length of dendrites in cells expressing either K_V_3.1b^WT^ or K_V_3.1b^R320H^ (Figure 4H). TEA did not prevent effects of K_V_3.1b^R320H^ on neurite growth compared to K_V_3.1b^WT^, suggesting that the defects introduced by the mutation were not simply due to altered potassium currents (Figure 4H). Consistent with this, blocking all K_V_3 family members with TEA had less severe effects on dendritic length than expression of K_V_3.1b^R320H^, indicating that blocking channel activity is less important for dendrite development than the presence of the disrupting mutation (Figure S6).

To investigate the contribution of other potassium channels blocked by TEA, we used a specific blocker for large‐conductance calcium‐activated potassium channels (BK channels), as these channels are dendritically expressed.^32^ We incubated neurons transfected with GFP with 100 mmol·L^–1^ IbTX,^33^ with or without TEA for 48 h. IbTX alone had no effect on dendritic length, and the application of IbTX with TEA resulted in a modest but nonsignificant reduction in dendritic length (Figure S7), suggesting that reduction was mainly mediated by reduced K_V_3 channel activity.

### 3.7 K_V_3.1b^R320H^ does not introduce gating pore currents

**FIGURE 5:**
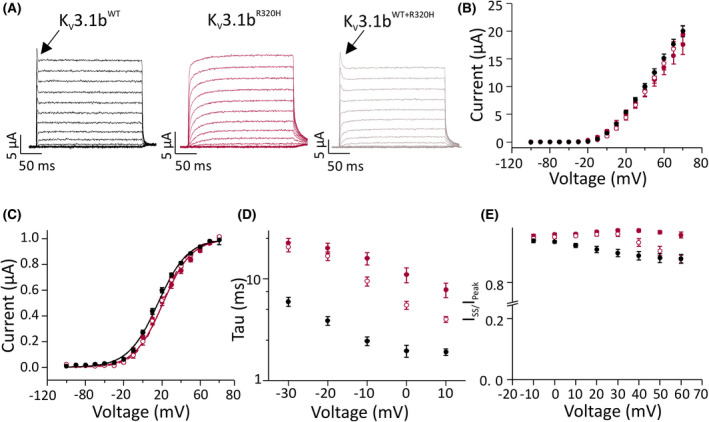
K_V_3.1^R320H^ channel properties in *Xenopus laevis* oocytes. (A) Representative current traces of K_V_3.1^WT^, K_V_3.1^R320H^, and K_V_3.1^WT+R320H^ channels in response to test voltage steps from −100 mV to +60 mV in 10mV increments. Holding voltage was −80 mV; tail voltage following the test pulses was −30 mV. (B–E) K_V_3.1^WT^ data are shown in black, K_V_3.1^R320H^ in solid red, and K_V_3.1^WT+R320H^ data in open red symbols. Data are mean ± SEM; K_V_3.1 ^WT^, *n* = 34; K_V_3.1^R320H^, *n* = 21; K_V_3.1 ^WT+R320H^, *n* = 13. (B) Mean current amplitude at the end of the test pulse is plotted against the test voltage. (C) Current at the beginning of the tail voltage step is plotted against the test pulse voltage and fitted with the Boltzmann equation. Black line represents the fit of the Boltzmann equation to the mean data for K_V_3.1^WT^, red line for K_V_3.1^R320H^ channels, and red line with open circles represents the fit for K_V_3.1 ^WT+R320H^ channels. Half maximal voltage of inactivation was .3 ± 2.4 mV, .4 ± 1.34 mV, and −4.9 ± 1.3 mV for homomeric mutant, simulated heterozygous condition, and wild‐type (WT) channels, respectively (*p* > .05, two‐way analysis of variance [ANOVA] followed by Bonferroni multiple comparison test). (D) Time constant of activation is plotted against the test voltage. At 0 mV, this was 20.1 ± 2.3 ms, 16.8 ± 1.7 ms, and 3.9 ± .4 ms for homomeric mutant, simulated heterozygous condition, and WT channels, respectively (*p* < .001 for WT vs. homomeric and simulated heterozygous mutant, two‐way ANOVA followed by Bonferroni multiple comparison test). (E) Fraction of inactivation is expressed as current at steady state (SS; end) of the pulse divided by the peak current of the pulse. At 60 mV, the remaining fraction was .96 ± .01, .88 ± .01, and .88 ± .01 for homomeric mutant, simulated heterozygous condition, and WT channels, respectively (*p* < .001 for homomeric mutant channel vs. simulated heterozygous or WT, two‐way ANOVA followed by Bonferroni multiple comparison test)

K_V_3.1b^R320H^ could disrupt dendritic development and survival by introducing a gating pore current.^19^ Gating pore currents are best detected in *Xenopus laevis* oocytes, where large currents are produced. In contrast to reported complete^4^ or partial^18^ reductions in expression in some experimental conditions, K_V_3.1b^R320H^ produced WT‐like current amplitude in our conditions (Figure 5A,B). Voltage dependence of activation also did not differ from the WT channels (Figure 5C). However, K_V_3.1b^R320H^ activation was slower than WT channels. The reduced rate of activation persisted in the simulated heterozygous condition with RNA for mutant and WT subunits injected at a 1:1 ratio (Figure 5D). Finally, WT channels showed a rapid but small decay in currents in response to pulses positive to 0 mV, and this inactivation was not seen in oocytes expressing K_V_3.1b^R320H^ (Figure 5E). Simulated heterozygous channels were inactivated at voltages more positive than WT channels. Overall, K_V_3.1b^R320H^ conferred dominant loss‐of‐function due to a reduced rate of channel activation.

**FIGURE 6:**
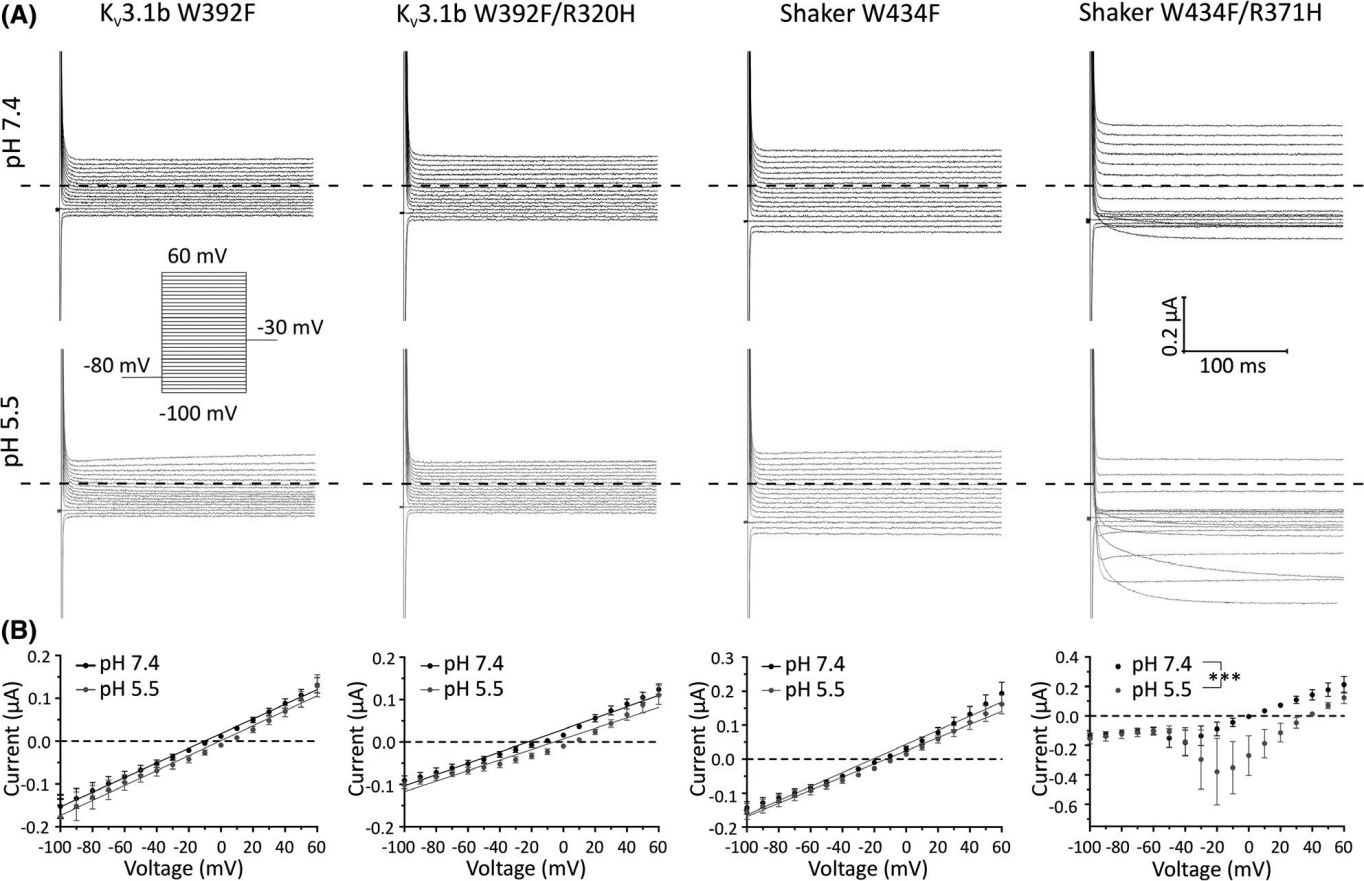
H^+^‐carried gating pore currents are not detectable for K_V_3.1b^R320H^. (A) Representative traces for K_V_3.1b and Shaker alpha‐pore mutants at pH 7.4 and 5.5. Currents were recorded from the same cells at different pHs. (B) Current amplitudes measured at pH 7.4 or pH 5.5 plotted against voltage for K_V_3.1b^W392F^ (*n* = 5), K_V_3.1b^W392F/R320H^ (*n* = 7), Shaker^W434F^ (*n* = 5), and Shaker^W434F^ ^/R371H^ (*n* = 5). K_V_3.1b^W392F/R320H^ did not produce measurable gating pore currents. R371H in Shaker^W434F^ introduces gating pore currents that are inward deflections from linear leak I/V relationships that are significantly larger at pH 5.5 than at pH 7.4 (area under the curve, ***p < .001, paired Student *t*‐test), indicating that they are carried by H^+^

The adverse effects of K_V_3.1b^R320H^ on neuronal development and viability may result from toxic H^+^ currents that leak through the VSD of the channel upon neuronal depolarization.^19^ To investigate the presence of these currents, we introduced a W392F mutation in the central pore to abolish K^+^ currents. The analogous pore mutation (W434F) in the *Drosophila* K_V_1 (Shaker) has been used to isolate gating pore currents from channels containing different S4 arginine mutants.^19^ We used Shaker^W434F^ carrying the analogous S4 arginine mutation, R371H, which produced robust depolarization‐activated currents that were enhanced at acidic pH, as a positive control for gating pore currents (Figure 6). Unlike these controls, neither K_V_3.1^W392F/ R320H^ channels, Shaker^W434F^, nor WT K_V_3.1^W392F^ produced voltage‐dependent currents differing from a linear leak at pH7.4 or 5.5 (Figure 6). These data suggest that gating pore currents do not underlie MEAK.

## 4 DISCUSSION

We have shown that the PME variant K_V_3.1b^R320H^ reduces neuronal excitability and, unexpectedly, impairs dendritic development in interneurons. A general reduction in K^+^ currents by TEA block does not completely reproduce K_V_3.1b^R320H^ dendritic phenotypes, suggesting mutation‐specific mechanisms. Although we confirm dominant negative effects on channel activity, gating pore currents do not appear to be a pathogenic mechanism. Defects in dendritic development due to K_V_3.1b^R320H^ may underlie MEAK pathology.

The fast activation and deactivation kinetics of K_V_3.1 channels are critical for supporting high‐frequency firing, and the major functional effect we observed was slowing of the activation kinetics. This slowing is consistent with an impaired ability of interneurons to support high‐frequency firing. As expression of mutant channels decreased frequencies below rates seen in GFP controls, these data cannot be explained by simple channel loss‐of‐function and indicate a dominant effect of the mutant on neurophysiology; however, immunolabeling interneurons at 14 DIV shows low endogenous K_V_3.1b channel expression. Firing phenotypes, although they can be strongly determined by K_V_3 channels,^34^, ^35^, ^36^ are modified by the balance of many channels,^36^ as well as by somatodendritic morphology. The developmental alterations caused by K_V_3.1b^R320H^ channels could contribute to reduced interneuronal excitability. Paradoxically, although dendrites were severely reduced, capacitance was not changed by K_V_3.1b^R320H^ expression; however, this may reflect limitations of space clamp of distal dendrites in our somatic recordings.

Our findings suggest a new role for K_V_3.1b^R320H^ in neurodevelopment, with mutant channels impairing dendritic outgrowth, even when expressed during early dendritic sprouting, rather than producing collapse. Our results are similar to the analogous R > H mutation in K_V_3.3 (K_V_3.3^R423H^), which causes spinocerebellar ataxia 13 (SCA13) and exhibits a similar slowing of channel activation.^37^ K_V_3.3^R423H^ also reduces dendritic length and compromises viability when expressed in Purkinje cells in vitro.^38^

K_V_3.1b^R320H^ showed a slower activation rate compared to WT channels, with unchanged voltage dependence of activation. Our findings are in agreement with a previous study that also used the b isoform.^18^ It appears that the effects of R320H differ depending on splice isoform, as a complete loss‐of‐function with a dominant alteration in the voltage dependence of activation was previously reported for K_V_3.1a^R320H^.^4^ Whereas a reduced current amplitude related to intracellular channel retention has also been reported for K_V_3.1b^R320H^ in HEK cells, we found no change in the current amplitude, instead observing robust expression in both oocytes and neurons. As with the analogous hK_V_3.3 mutant (K_V_3.3^R423H^), K_V_3.1b^R320H^ does not conduct gating pore currents.^37^

Blocking all K_V_3 conductances with TEA during development only caused a mild reduction in dendritic length compared to the overexpression of K_V_3.1b^R320H^, suggesting that the detrimental effects of K_V_3.1b^R320H^ on dendrites are not due to a simple decrease in high‐voltage‐activated potassium currents. This suggests there may be nonconducting effects of the mutation leading to the dendritic deficits. Overexpression of K_V_3.1b^WT^ had no effect on development, indicating that increasing high‐voltage‐activated potassium currents does not promote excessive neurite development. However, overexpressed WT channels could rescue the effect of TEA on dendritic length; either K_V_3.1 currents were not completely blocked by TEA, or WT channels also have nonconducting roles impacting dendritic growth.

*KCNC1* variants are associated with early infantile onset of symptoms in DEE (A421V), supporting a developmental role for K_V_3.1 channels. In the rodent brain, K_V_3.1b expression increases throughout early postnatal development and into adulthood, corresponding with the maturation of electrical circuits.^11^ K_V_3.1 channels have also been implicated in cell migration and the proliferation and differentiation of neural precursor cells.^39^, ^40^ The early transient expression of K_V_3.1 channels suggests distinct nonconducting roles for these channels before neurons become electrically excitable. The extended C‐terminus of the closely related K_V_3.3 is important for actin binding and cell survival,^41^ and a similar role may be played by the C‐terminus of K_V_3.1. K_V_3.1b^R320H^ also appears to reduce neuronal viability, a process that may be linked to progressive cerebellar atrophy and ataxia in patients.^5^ Maladaptive remodeling of dendrites of Purkinje cells in response to dendritic hyperexcitability has been proposed as a possible mechanism in K_V_3.3‐associated SCA13, changes that may ultimately contribute to neurodegeneration.^42^ In SCA1/2, reduced dendritic K_V_3.2 and K_V_3.3 conductances appear as an “acquired channelopathy.”^43^, ^44^ Further work is needed to assess how these processes apply to cortical interneurons and MEAK.

Deficits in neuronal development are suggested for other forms of PME that are phenotypically similar to MEAK and prominently feature ataxia. In fly models of North‐Sea PME, mutations in the Golgi SNARE protein *GOSR2* (OMIM # 614018) have been shown to impair dendritic outgrowth.^45^ Haploinsufficiency of prickle planar cell polarity protein 1 (*PRICKLE1*; OMIM # 612437) also causes PME. *Prickle1* regulates neurodevelopment,^46^ and its knockdown has been shown to result in deficits in dendritic and axonal outgrowth.^47^ If such morphological deficits could be reversed or stabilized, new treatment avenues could emerge for these severe, treatment‐resistant epilepsies. In the case of MEAK, positive modulators of K_V_3.1 function may have clinical utility in normalizing firing rates,^18^, ^48^, ^49^ but whether they also hold value in preventing relentless disease progression depends on whether the dendritic deficits are due to nonconducting roles of K_V_3.1b.

One limitation to this study is that we focused on cortical interneurons in vitro, but the dominant negative effects of K_V_3.1b^R320H^ on other K_V_3 family members will likely depend on the expressing cell type, with the possibility of neurons expressing different K_V_3 family members responding differently to K_V_3.1b^R320H^. Nonconducting roles of K_V_3.1b may also be dependent on cell type and developmental stage. A knockin mouse model would be better positioned to answer these questions, as well as to model the heterozygous condition, as seen in patients. Although we used a WT background to model heterozygosity, we acknowledge that a limitation of our study is that neurons in vitro do not express high amounts of the endogenous channel.

Overall, our results show a loss‐of‐function, dominant negative effect of K_V_3.1b^R320H^ on neuronal firing and an additional pronounced suppression of dendritic development. The effects on dendrites suggest MEAK, and more broadly other forms of PME, may represent a group of dendritopathies.

## CONFLICT OF INTEREST

None of the authors has any conflict of interest to disclose.

## Supporting information

Supplementary Material
