# Supplementary material for: Progressive myoclonus epilepsy KCNC1 variant causes a developmental dendritopathy
Source: Epilepsia. 2021 Mar 18;62(5):1256–67. doi: 10.1111/epi.16867 (PMC8436768; doi:10.1111/epi.16867)
Supplement: Supplementary file 1 — Supplementary Material [file EPI-62-1256-s001.docx]

**Supplementary Materials**

**Extended Materials and Methods**

### ***Animals and ethics.***

### Animal care and experimental procedures were carried out in accordance with the UK Animals (Scientific Procedures) Act 1986.

### ***Molecular biology***

Mutations were introduced using PCR (QuikChange, Agilent Technologies) and mismatch mutagenic primers (Eurofins Genomics). The R371H mutation was introduced into the non-conducting (W434F)^1^, fast-inactivation removed (IR, Δ6–46)^2^ Shaker H4 K^+^ channel (accession: NM_167595.3) that was a gift of Fredrik Elinder, Linkoping University (for primers see Supplementary methods table). Human *KCNC1* CDS encoding the b isoform (accession: NM_001112741.2) was codon optimised for expression in murine cells using GeneOptimizer software and commercially synthesised by GeneArt (Thermo Fisher Scientific). The c.959G>A PME mutation, which results in the substitution of arginine 320 with histidine, was introduced by site-directed mutagenesis. Shaker and Kv3.1b sequences were aligned using the online tool, *Clustal Omega,* to identify W392F in Kv3.1b as equivalent to the W434F non-conducting α-pore mutation in Shaker ^3^. WT and mutant *KCNC1* and Shaker transgenes were subsequently cloned into a pSGEM expression vector (a gift of Dr M.Hollman, Göttingen Germany). The sequence integrity for the entire length of all transgenes was confirmed by Sanger sequencing (Source Bioscience).

Identical vectors used in transfection and lentiviral synthesis were constructed using a pCCL lentiviral transfer plasmid ^4^. Transgene expression was restricted to interneurons using the mouse Dlx5/6 (mDlx) enhancer/ promoter ^5^. A DNA fragment comprised of the mDlx5/6 promoter, dscGFP and a Thosea asigna virus 2A (T2A) self-cleaving signal peptide sequence was commercially synthesised (GeneArt, *Invitrogen*) and subcloned into pCCL. WT and mutant Kv3.1b transgenes were subsequently subcloned downstream and in frame with the T2A sequence. All plasmids were sequenced before use (Source Bioscience).

## *Channel expression in Xenopus oocytes*

Oocytes were removed from *Xenopus laevis* toads. Stage V-VI oocytes were enzymatically defolliculated in oocyte Ringer’s solution that contained (in mM): 82.5 NaCl, 2 KCl, 5 HEPES, 1 MgCl_2_ at pH 7.5-7.6 with 2mg / mL of Collagenase A. Oocytes were preserved in Modified Barth’s Solution containing (in mM): 87.1 NaCl, 1 KCl, 10 HEPES, 1.7 MgSO_4_, 0.9 NaNO_3_, 2.4 NaHCO_3_ and 0.9 CaCl_2_, pH 7.4) at 14-18°C, which was supplemented with penicillin (50 U / mL) and amikacin (100 µg / mL) following oocyte injection.

Copy RNA (cRNA) was synthesised in an *in vitro* T7 transcription reaction (mMESSAGE mMACHINE™, Ambion) using linearised template DNA containing Kv3.1b (NheI) or Shaker transgenes (HindIII). Oocytes were injected with a total of 2.5 ng of cRNA (Nanoject, Drummond). When studying Kv3.1 heteromers, Kv3.1b^WT^ and Kv3.1b^R320H^ cRNAs were co-injected at a 1:1 mass ratio for a total of 2.5 ng of cRNA.

***Electrophysiological recordings of oocytes***

Two-electrode voltage clamp **(**TEVC) oocyte recordings were obtained 48-72 hours after oocyte injection. Signals were filtered at 1 kHz using a Bessel low-pass filter, acquired using GeneClamp 500B amplifier (Axon Instruments), digitised using a Digidata 1200 and sampled at a rate of 5 kHz using pCLAMP™ (Molecular Devices) software. For all recordings, electrodes were filled with 3M KCl solution (pH 7.4) and had a tip resistance of 0.1 - 0.5 MΩ. The bath solution contained: 120 mM Na^+^-methanesulfonate (NaMeSO_4_)_,_ 120 mM CH_3_SO_3_Na, 1.8 mM CaSO_4_ and 10 mM HEPES, at pH 7.4. For gating pore current recordings, oocytes were perfused first with NaMeSO_4_ solution at pH 7.4 followed by NaMeSO_4_ at pH 5.5.

Oocytes were held at a potential of -80 mV. The voltage protocol used to measure whole cell K^+^ currents, consisted of 250 ms test voltage steps ranging from -100 mV to +60 mV (Δ10 mV), followed by a tail step to -30 mV for 250 ms, carried out in the presence of the -*P*/4 leak subtraction protocol. Voltage dependence of activation was assessed by measuring the current amplitude at the beginning of tail voltage step, plotting this against the test voltage and fitting with the Boltzmann equation. The time course of activation was studied by fitting an exponential curve to the current traces recorded in response to test pulses following the settling of capacitive transients.

Gating pore current recordings were carried out in the absence of leak subtraction protocols, using the same voltage protocol described above. The voltage dependence of gating pore currents was analysed by plotting the mean current during the last 200 ms of the test pulse against the voltage. Linear leak conductance was estimated by fitting a straight line (y = mx + b) to the current-voltage data at the most negative test voltages. IV plots were fit with linear regression, and for channels with proton leaks only points outside of the region where the proton currents were seen were used to produce the fit. Area under the curve (AUC) was calculated subtracting the linear component.

***Lentiviral production***

VSVg pseudotyped second-generation HIV-1 based lentiviral particles were generated using previously described methods ^6^, with the following amendments: the mass ratio of transfer: packaging: envelope vectors was: 1.25 : 2 : 1 and the viral supernatant was concentrated by ultracentrifugation at 48,298 g for 2 hr at 4°C without a sucrose gradient. Functional lentiviral titres were calculated using genomic DNA extracted from transduced HEK cells using Lenti-X Provirus Quantitation Kit (Takara Bio USA).

***Primary neuronal culture***

Primary cortical neuronal cultures were prepared from postnatal day 0-1 C57BL/6J mouse pup cortices according to a previously described protocol ^7,8^. Cortical cultures were transfected at 4-5 days in vitro (DIV) using magnetofection™ ^9^. Neurons were incubated on a magnet for 10 min at 37°C in Neurobasal A (Gibco) with 100 µL Opti-MEM (Gibco) reduced serum medium containing 0.1 µg DNA and 1 µL of NeuroMag (OZBiosciences) and then for a further 30 min without the magnet. After transfection, the culture media was completely replaced with preconditioned media to aid recovery, which, when assessing the effect of Kv3 blockade on dendritic growth, was supplemented with tetraethylammonium chloride (1mM in water, Sigma) or 100 mM iberiotoxin (IbTX). Lentiviral transduction of cultures was carried out at 1-2 DIV.

***Immunocytochemistry***

Cortical neurons were fixed in 4% paraformaldehyde in PBS (Affymetrix, USB) for 15 min and then washed 3X for 5 min in 1X PBS. Neurons were permeabilised and non-specific binding sites blocked in 5% Normal Goat Serum (NGS) in PBS with 0.1% TritonX-100 (PBS-T) for 30 min, prior to incubation with the relevant primary antibody in 2% NGS in PBS-T overnight at 4°C. Neurons were washed 3X in PBS and then incubated with Alexa Fluor conjugated 555 or 647 antibodies in 2% NGS in PBS-T, for 1 hr (all 1:1000, Invitrogen). Neurons were washed 3X in PBS and mounted onto glass slides (VWR) using Prolong^TM^ Gold antifade reagent with 4',6-diamidino-2- phenylindole (DAPI) (Invitrogen). Images were acquired using an inverted LSM 710 (Zeiss) confocal laser scanning microscope (ZEN software, 2009) with X20 objective or X40 or X63 EC Plan-Neofluar oil-immersion objective (Zeiss). The following primary antibodies were used: rabbit α-K_V_3.1b (1:500, Alomone Labs), SMI-31 (1:1000, BioLegend), Guineapig α-MAP2 (1:1000, Synaptic Systems). Apart from where specified, all incubations were carried out at room temperature.

***TUNEL staining***

TUNEL assays were performed using the 594CF^TM^ Dye TUNEL Assay Apoptosis Detection Kit (Biotium) according to the manufacturer’s protocol.

***Image analysis***

Image analysis was performed using Image J (Version 1.51 u). Neurites were semi-automatically traced using the NeuronJ plugin ^10^. Sholl analysis was performed on traced dendrites using a step size of 1μm.

***Electrophysiological recordings of neuronal cultures***

Whole cell current-clamp electrophysiological recordings of cortical neurons were carried out at 14 - 16 DIV. Electrophysiological signals were acquired using a MultiClamp 700A amplifier (Axon Instruments, Molecular Devices) and digitised using a CED Power 1401-3A digital-to-analogue converter. Signals were filtered at 10 kHz using a Bessel filter and sampled at 20 kHz. Recording electrodes were microfabricated from thin-walled borosilicate glass and had a resistance of 4-6 MΩ. Voltage-clamp was used to obtain the gigaohm seal and to obtain a whole-cell recording. Cells were held at -70 mV in current clamp configuration. Recordings were carried out at 32°C with continuous oxygenated perfusion, at a rate of 5 ml / min. The internal solution contained (in mM): 148 K-gluconate; 4 NaCl; 1 MgSO_4_; 0.02 CaCl_2_; 0.1 BAPTA; 15 glucose; 5 HEPES; 3 ATP and 0.1 GTP (-15 mV liquid junction potential). The external solution contained (in mM): 119 NaCl, 25 NaHCO_3_, 11 glucose, 2.5 KCl, 1.25 NaH_2_PO_4_, 2.5 CaCl_2_ and 1.3 MgCl_2_. Synaptic transmission was blocked using 50 µM DL-2-Amino-5-phosphonovaleric acid (APV) (Tocris /ChemCruz), 10 µM NBQX (Tocris Bioscience) and 30 µM PTX (Tocris Bioscience). Transduced neurons were identified by GFP fluorescence. Recordings were discarded if the unadjusted resting membrane potential was < - 55 mV, if the holding current at - 70 mV > - 200 pA or if the bridge balance was > 10 MΩ. A current step protocol was used to trigger APs by injecting currents ranging from –20 pA to +300 pA (Δ10 pA) for 1 s. To investigate cellular firing frequencies (from 10 to 100 Hz), neurons were injected with 10 current pulses at 110% of the AP current threshold using a 5 ms stimulus. The current threshold was found by iteratively injecting neurons with 5 ms depolarising steps (Δ10 pA) until an action potential was fired.

## *Data analysis*

Electrophysiological signals were analysed using ClampFit software (Clampex, Molecular Devices) or a custom Python script, and graphs were created using Prism (GraphPad Software, Inc., CA, USA). The spike success rate was calculated as the percentage of APs successfully elicited for 10 stimuli, averaged over three sweeps. An AP was defined as a spike crossing 0 mV with a rising slope > 20 V/s. AP shape parameters were extracted using a phase-plane plot implemented in the Python script ^11^. The rising slope was defined as the highest V/s value; the voltage threshold as the first voltage point where the derivate was > 20 V/s; AP peak as the highest voltage value (mV) reached and AP half width as the Δt half-way between the AP peak and the voltage threshold. AP analysis was carried out on the first AP elicited by 1s square pulse current injections. Passive properties were calculated as following: input resistance - as the slope of a line passing by 3 points calculated at -20 pA, -10 pA and 10 pA current injection as ΔV / current injected; capacitance as tau / input resistance; where tau was calculated by fitting a single exponential between the baseline and the plateau voltage obtained by a -20 pA current injection; resting membrane potential was calculated without current injection. All experiments and data analysis were carried out with the experimenter blinded to the lentiviral vector transduced.

***Statistics***

Data are plotted as scatter plots, representing single data points. Box plots show the mean (+), median (middle line), percentiles (25% - 75%) and max / min points where represented. The statistical analysis performed is shown in each figure legend. Deviation from normal distributions was assessed using a D’Agostino-Pearson’s test, and the F-test was used to compare variances between more than two sample groups. One-way ANOVA was used to compare three groups and was followed by a post-hoc test for functional analysis. To compare two groups at different time points a two-way repeated measure ANOVA, followed by a post-hoc test for functional analysis, was used. Statistical analysis was carried out using Prism (GraphPad Software, Inc., CA, USA) with significance set at p < 0.05.

**Supplementary References**

1. Perozo E, Mackinnon R, Bezanilla F, Stefanis E. Gating currents from a nonconducting mutant reveal open-closed conformations in Shaker K + channels. Neuron. 1993;11:353–8.

2. Hoshi T, Zagotta W, Aldrich R. Biophysical and molecular mechanisms of Shaker potassium channel inactivation. Science. 1990;250:533–8.

3. Perozo E, MacKinnon R, Bezanilla F, Stefani E. Gating currents from a nonconducting mutant reveal open-closed conformations in Shaker K+ channels. Neuron. 1993;11:353–8.

4. Dull T, Zufferey R, Kelly M, Mandel RJ, Nguyen M, Trono D, et al. A Third-Generation lentivirus vector with a conditional packaging system. J Virol. 1998;72:8463–71.

5. Dimidschstein J, Chen Q, Tremblay R, Rogers S, Saldi G-A, Guo L, et al. A viral strategy for targeting and manipulating interneurons across vertebrate species. Nat Neurosci. 2016;19:1743–9.

6. Snowball A, Chabrol E, Wykes R, Shekh-Ahmad T, Cornford J, Lieb A, et al. Epilepsy gene therapy using an engineered potassium channel. J Neurosci. 2019;39:3159–69.

7. Cano-Jaimez M, Tagliatti E, Mendonca PRF, Nicholson E, Vivekananda U, Kullmann DM, et al. Preparation of dissociated mouse primary neuronal cultures from long-term cryopreserved brain tissue. J Neurosci Methods. 2020 Jan 15;330.

8. Tagliatti E, Bello O, Mendonça PRF, Kotzadimitriou D, Nicholson E, Coleman J, et al. Synaptotagmin 1 oligomers clamp and regulate different modes of neurotransmitter release. Proc Natl Acad Sci U S A. 2020;117:3819–27.

9. Mykhaylyk O, Antequera YS, Vlaskou D, Plank C. Generation of magnetic nonviral gene transfer agents and magnetofection in vitro. Nat Protoc. 2007;2:2391–411.

10. Meijering E, Jacob M, Sarria J, Steiner P, Hirling H, Unser M. Design and validation of a tool for neurite tracing and analysis in fluorescence microscopy images. Cytometry. 2004;58A:167–76.

11. Colasante G, Qiu Y, Massimino L, Di Berardino C, Cornford J, Snowball A, et al. In vivo CRISPRa decreases seizures and rescues cognitive deficits in a rodent model of epilepsy. Brain. 2020;143:891–905.

**Table 1: Primers used in site-directed mutagenesis**

| **Primer** | **Sequence (5’🡪3’)** |
| --- | --- |
| Shaker362-F | GTCCTTGGCAATATTACACGTGATACGATTAGTTCG |
| Shaker362-R | CGAACTAATCGTATCACGTGTAATATTGCCAAGGAC |
| Shaker371-F | CGATTAGTTCGAGTATTTCACATATTTAAGTTATCTAGGC |
| Shaker371-R | GCCTAGATAACTTAAATATGTGAAATACTCGAACTAATCG |
| Kv3.1W392-F | CATCCCCATCGGATTTTTTTGGGCCGTCGTGACC |
| Kv3.1W392-R | GGTCACGACGGCCCAAAAAAATCCGATGGGGATG |
| Kv3.1959G.A-F | TCGTGCGGATCCTGCACATCTTCAAGCTGAC |
| Kv3.1959G.A-R | GTCAGCTTGAAGATGTGCAGGATCCGCACGA |

**Supplementary Figures**

**
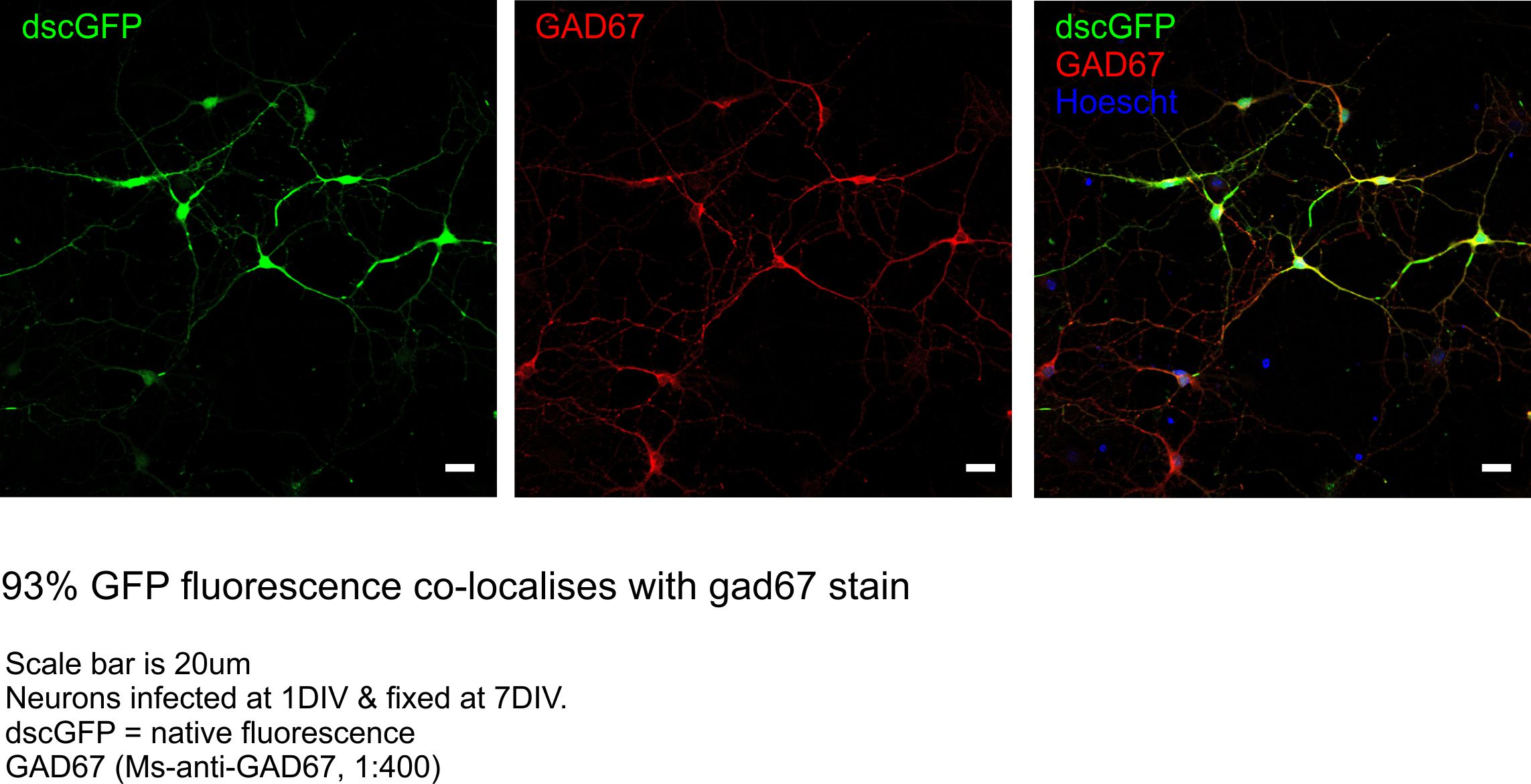
**

***Figure S1. High targeting specificity of interneurons in culture using the mDlx 5/6 promoter/enhancer.*** *Representative immunofluorescent images of GFP and GAD67 colocalisation in murine cortical neuronal cultures at 7 DIV, following transduction with a lentivirus expressing dscGFP from the mDlx5/6 promoter. 93 ± 1.9% of transduced (GFP+) neurons were found to be positive for GAD67 immunolabelling, following analysis of 10 regions of interest. Scale bar is 20 μm. GAD67: glutamate decarboxylase 67, dscGFP: destabilised copGFP.*


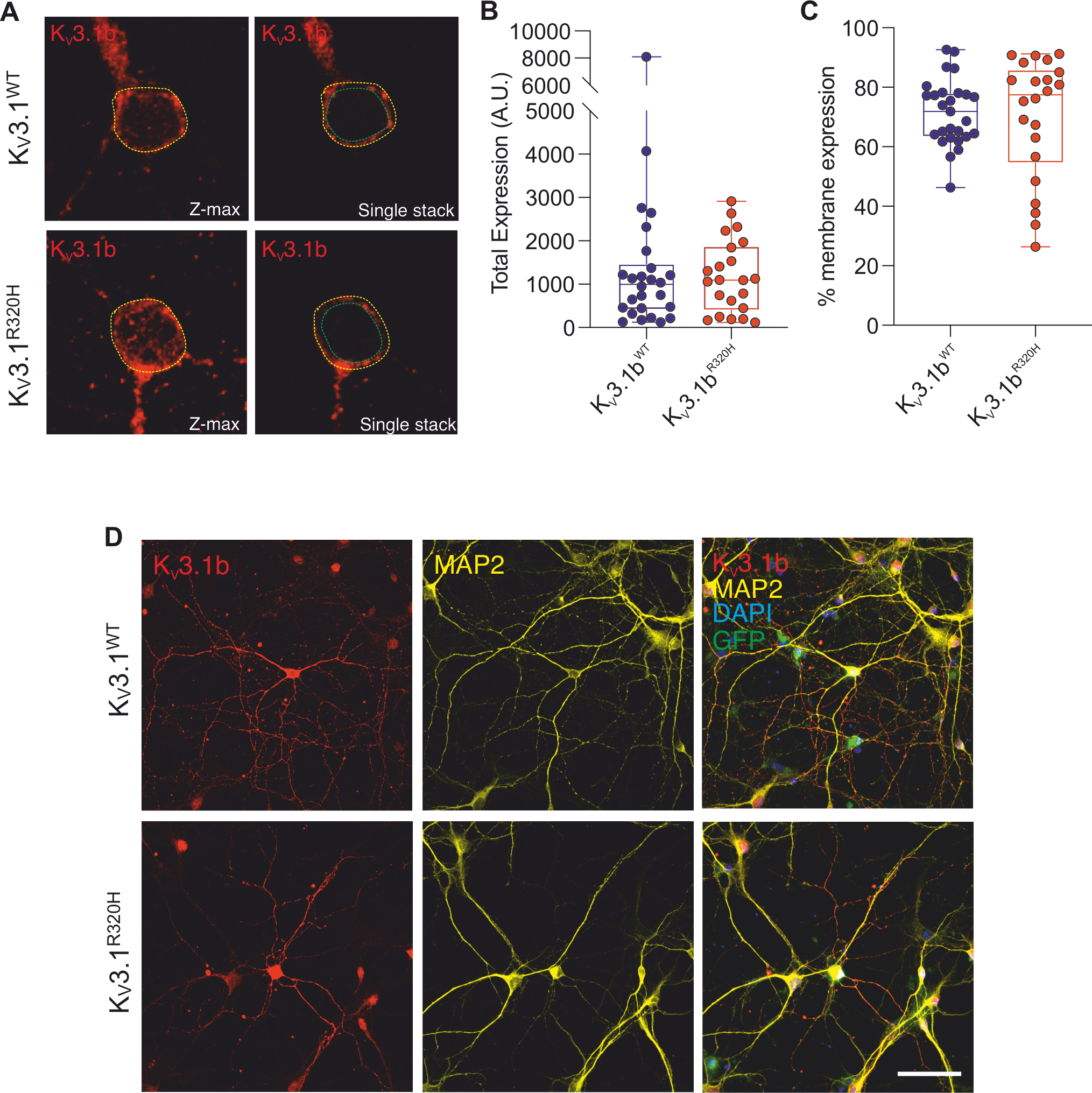


**Figure S2. K_V_3.1^R320H^ expression is comparable to K_V_3.1^WT^.** A) Representative immunofluorescent images of somatically expressed K_V_3.1b in cortical interneurons 14 days post-lentiviral transduction with K_V_3.1^WT^ or K_V_3.1^R320H^. Yellow dotted lines show the area measured to find the total fluorescence for the cell body (left panel) and green dotted lines delimit the area measured for the membrane fraction (single middle stack, right). B) Quantification of total expression in the cell body. C) Quantification of the membrane fraction (% total area- green dotted area). No significant differences were observed. Each data point represents one cell. Data are from two independent samples. D) WT and mutant K_V_3.1b expression in the dendrites. Representative immunofluorescent images of interneurons overexpressing K_V_3.1b at 7 DIV. Scale bar is 50 μm.

**
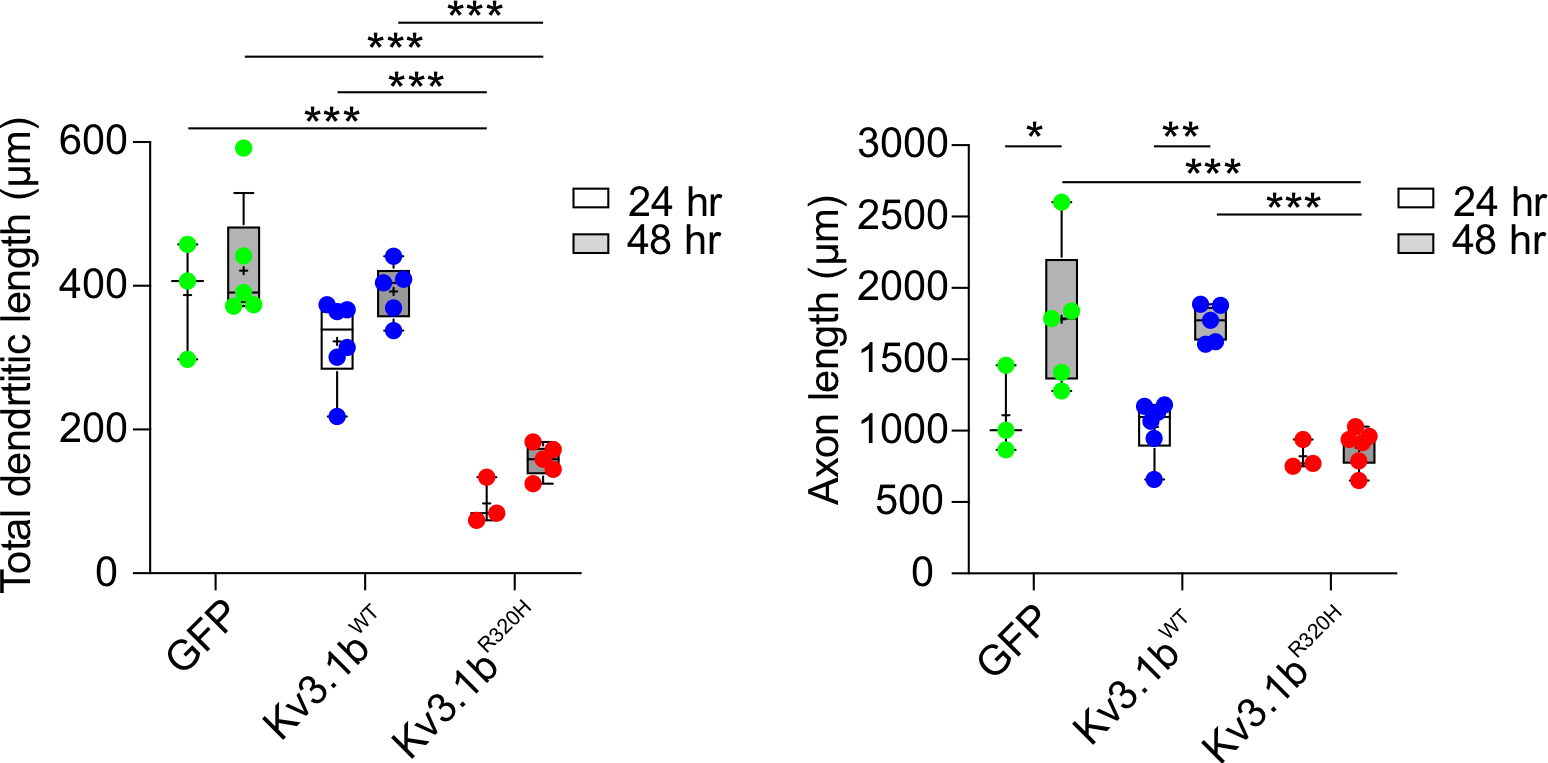
**

**Figure S3. Kv3.1b^R320H^ impairs neurite outgrowth.** Primary murine cortical neuronal cultures were magnetofected at 4 DIV with plasmids delivering GFP only or Kv3.1b variants. Neurites were traced and measured 24 and 48 hr post-transfection. *p<0.05, **p<0.01, ***p<0.001; Two-way ANOVA followed by Bonferroni multiple comparisons test.

**
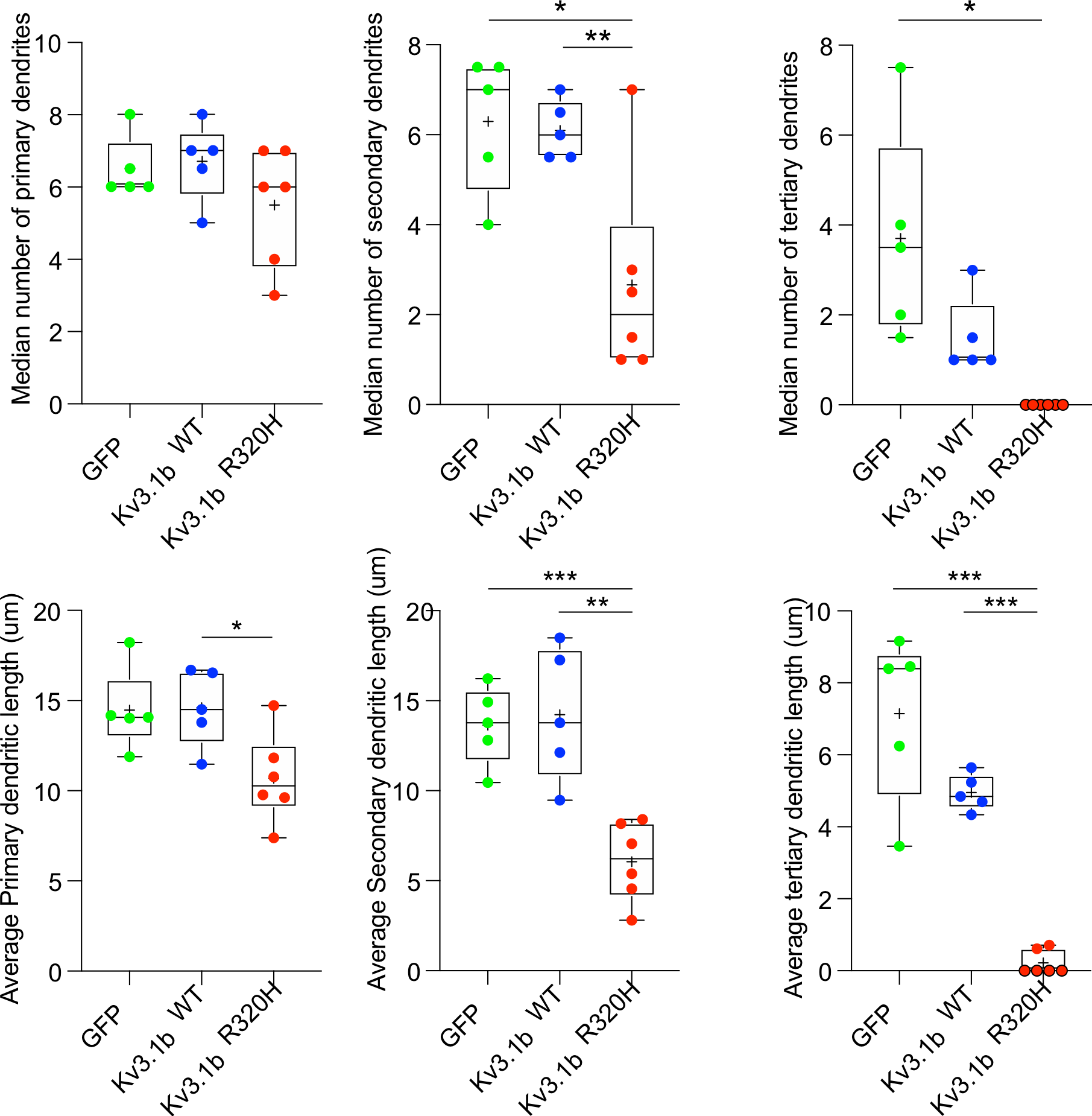
**

***Figure S4. Dendritic analysis of neurons transfected with Kv3.1b channel variants at 6 DIV.*** *Additional parameters of neurons transfected with either GFP, Kv3.1^WT^ or Kv3.1^R230H^. *p<0.05, **p<0.01, ***p<0.001; One-way ANOVA followed by Bonferroni’s multiple comparisons test.*


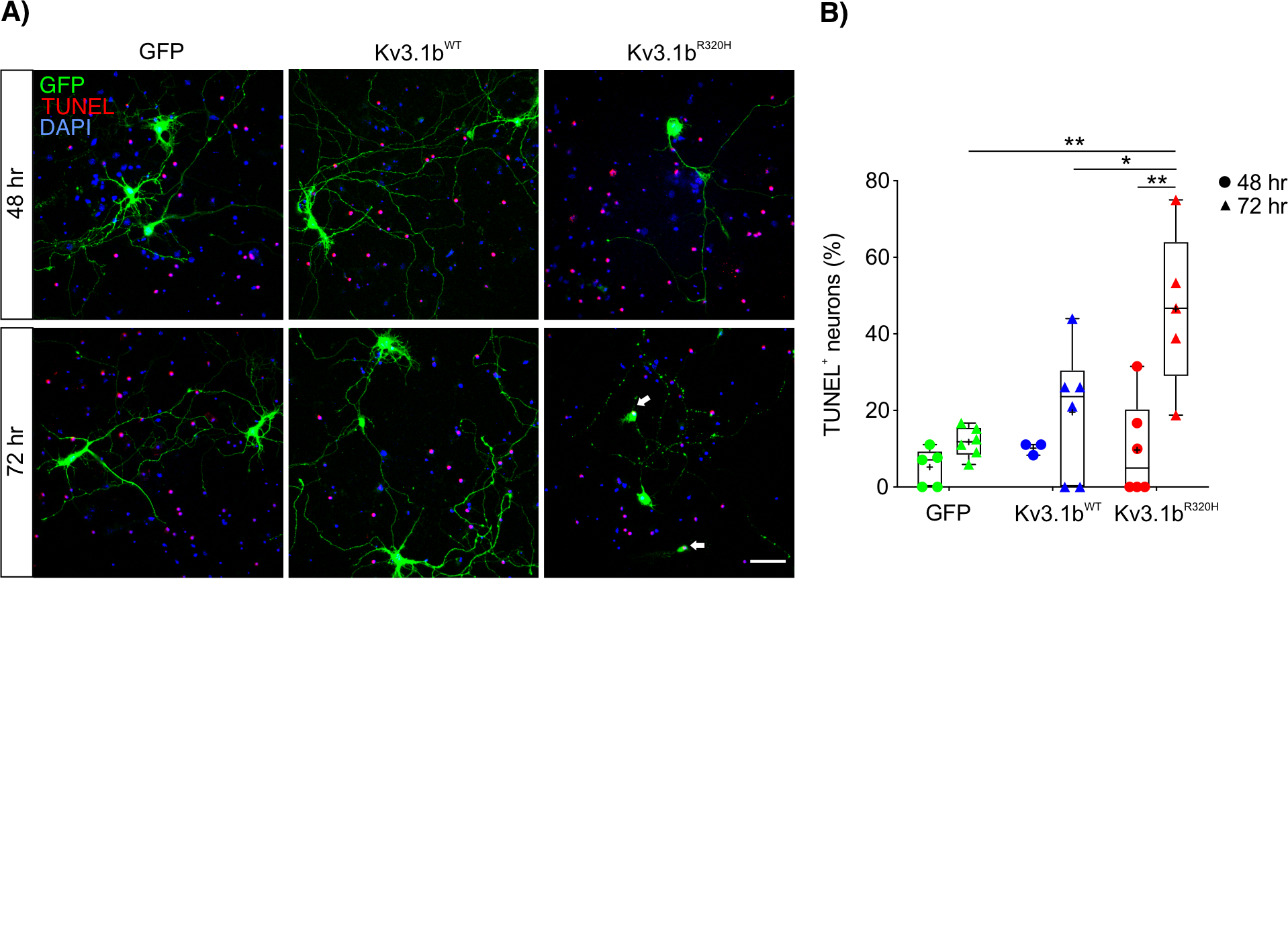


**Figure S5. Acute developmental expression of Kv3.1b^R320H^ channels reduces neuronal viability. A)** Representative images. Cortical neurons were magnetofected with plasmids delivering GFP or Kv3.1b channel variants at 4 DIV and a TUNEL assay performed after 48 or 72 hr (6 DIV and 7 DIV). **B)** Kv3.1b^R320H^ neurons are still viable after 48 hr of channel expression (**48 hr:** GFP vs. Kv3.1b^R320H^: p > 0.99; WT vs. Kv3.1b^R320H^: p > 0.99; GFP vs. Kv3.1b^WT^: p > 0.99). 72 hr of Kv3.1b^R320H^ channel expression significantly increases the percentage of transfected neurons with TUNEL positive nuclei with respect to Kv3.1b^WT^ and GFP controls (**72 hr**: GFP vs. Kv3.1b^R320H^: p = 0.0022; WT vs. Kv3.1b^R320H^: p = 0.029; GFP vs. Kv3.1b^WT^: p > 0.99). Neurons die between 48 and 72 hr of Kv3.1b^R320H^ channel expression (**48 vs. 72 hr**: Kv3.1b^R320H^: p = 0.001; Kv3.1b^WT^: p > 0.99; GFP: p > 0.99). Two-way ANOVA with Bonferroni’s multiple comparisons test. Data are from two independent neuronal preparations.


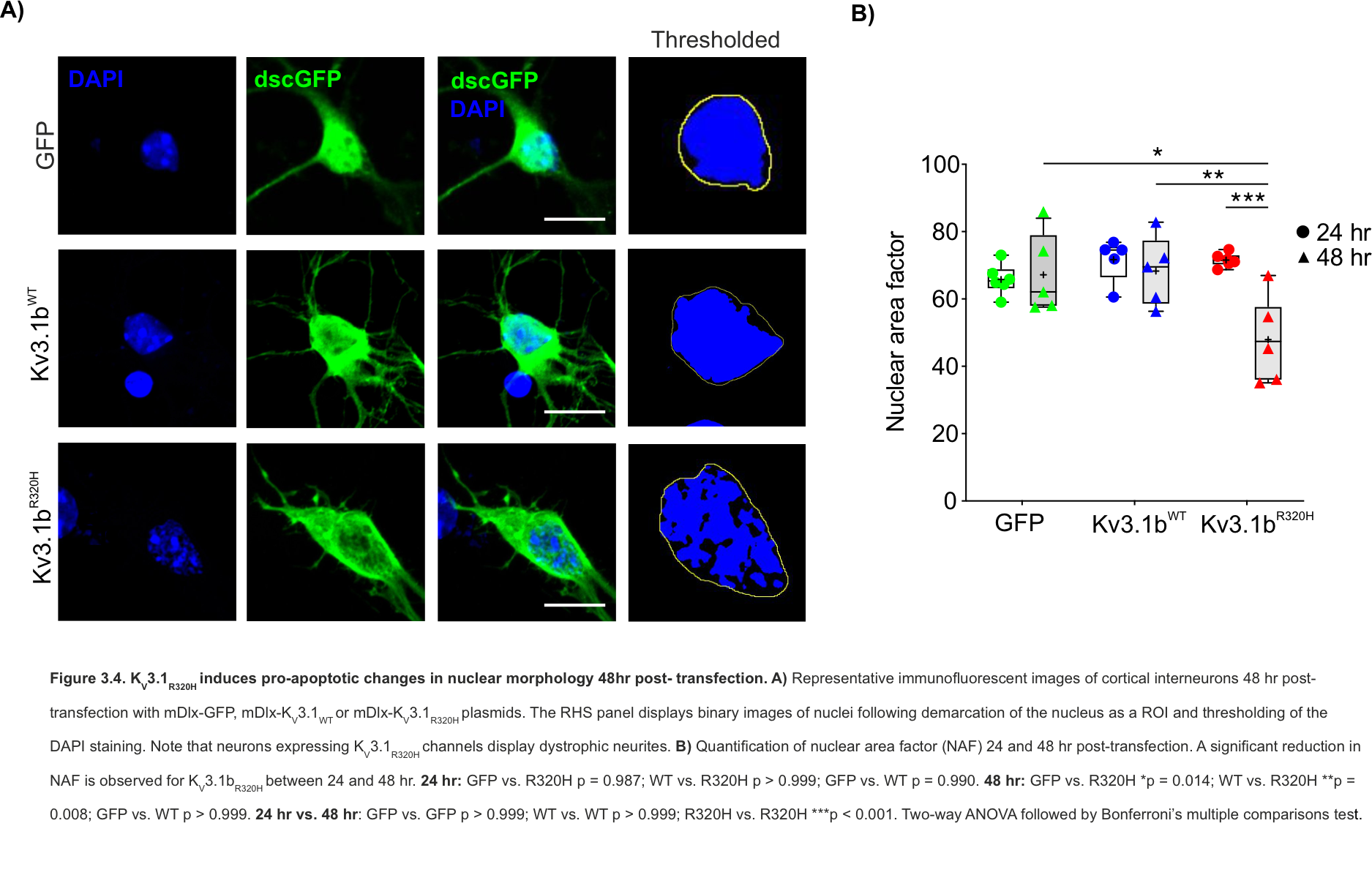


**Figure S6. Kv3.1^R320H^ induces pro-apoptotic changes in nuclear morphology 48hr post- transfection.** A) Representative immunofluorescent images of cortical interneurons 48 hr post- transfection with GFP, Kv3.1^WT^ or Kv3.1^R320H^. The RHS panel displays binary images of nuclei following demarcation of the nucleus as a ROI and thresholding of the DAPI staining. Note that neurons expressing Kv3.1^R320H^ channels display dystrophic neurites. B) Quantification of nuclear area factor (NAF) 24 and 48 hr post-transfection. Significantly reduced NAF for Kv3.1b^R320H^ between 24 and 48 hr. 24 hr: GFP vs. Kv3.1b^R320H^ p = 0.987; WT vs. Kv3.1b^R320H^ p > 0.999; GFP vs. WT p = 0.990. 48 hr: GFP vs. Kv3.1b^R320H^ p = 0.014; WT vs. Kv3.1b^R320H^ p = 0.008; GFP vs. WT p > 0.999. 24 hr vs. 48 hr: GFP vs. GFP p > 0.999; WT vs. WT p > 0.999; Kv3.1b^R320H^ vs. Kv3.1b^R320H^p < 0.001. Two-way ANOVA followed by Bonferroni’s multiple comparisons test. Data are from two independent neuronal preparations.


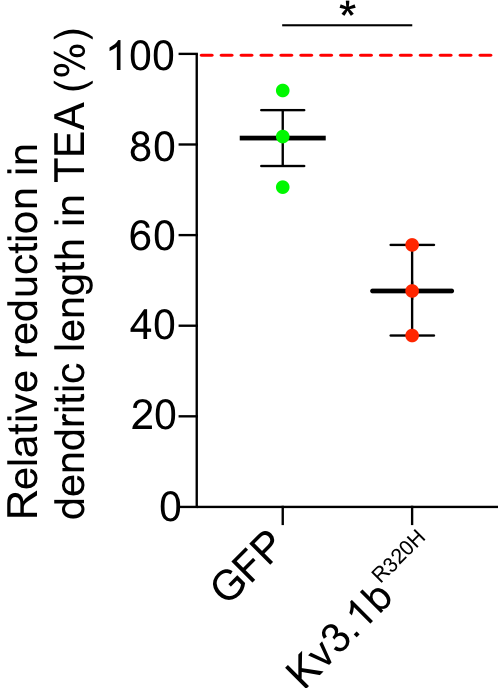


***Figure S7. Kv3.1b^R320H^ expression has more of a severe effect on dendritic development than TEA****. Cortical neuronal cultures were transfected at 5 DIV with GFP or Kv3.1b^R320H^ and treated with 1mM TEA. Dendrites were traced and measured 24 hr later (6 DIV) to find the total dendritic length. Neurons expressing Kv3.1b^R320H^ have significantly reduced dendritic length compared to GFP neurons treated with TEA only (p = 0.016, Student’s t-test).*

**
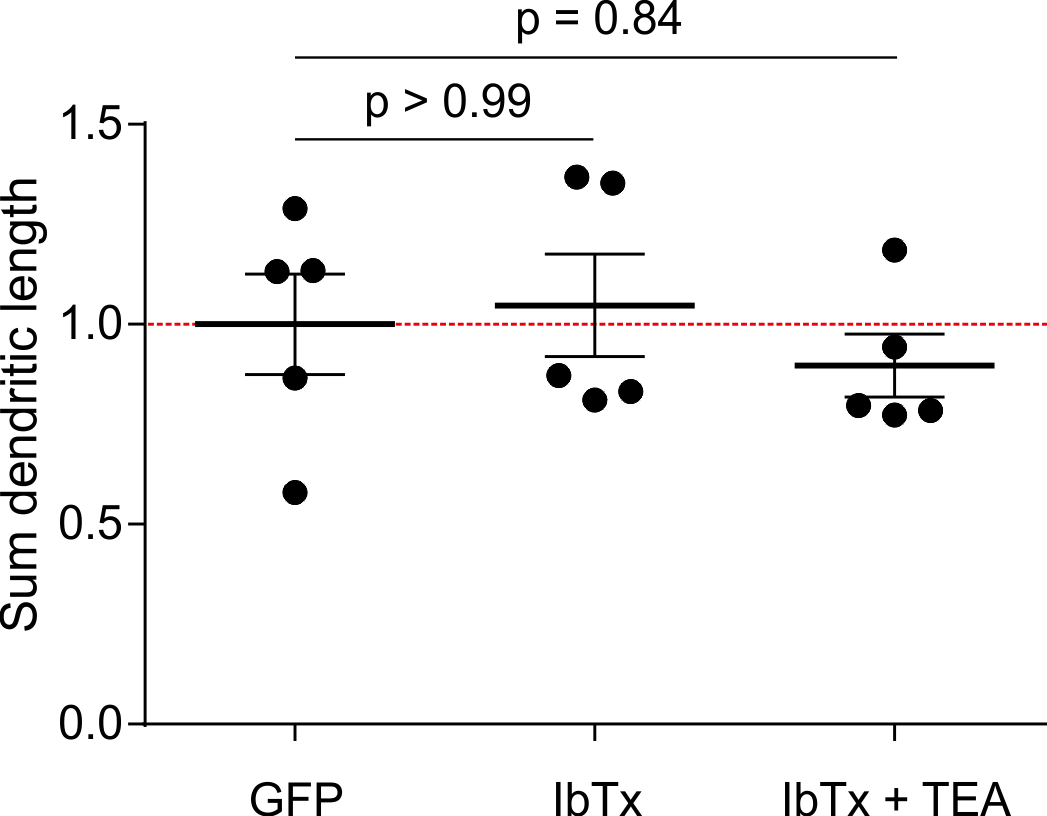
**

**Figure S8. Blockade of BK channels in developing cortical neurons has no effect on dendritic length.** Primary cortical cultures were transfected with mDlx-GFP at 5 DIV and were treated with 100 mM IbTx only, or IbTx + 1mM TEA, for 48 hr. Interneurons were identified by GFP fluorescence and dendrites were traced and measured at 7 DIV to find the total dendritic length, which was then normalised to the average total dendritic length for untreated controls (GFP). IbTx had no significant effect on dendritic length compared to untreated controls (p >0.99, one-way ANOVA, followed by Bonferroni’s multiple comparisons test) and IbTx treatment in combination with TEA resulted in a non-significant trend towards a reduction in dendritic length (p = 0.84). Data are from two independent neuronal preparations, where ‘n’ is the average of one coverslip.

**Supplementary Table 1. Active and passive properties of neurons expressing K_V_3.1b channels**

| **Property** | **GFP (n = 12)** | **K_V_3.1b^WT^ (n = 8)** | **K_V_3.1b^R320H^ (n = 6)** |
| --- | --- | --- | --- |
| Resting membrane potential (mV) | -60.2 ± 2.1 | -54.5 ± 1.6 | -58.0 ± 5.7 |
| Input Resistance (MΩ) | 301.0 ± 53.3 | 241.1 ± 29.5 | 329.3 ± 118.5 |
| Capacitance (pF) | 135.2 ± 22.4 | 101.7 ± 18.4 | 111.3 ± 15.1 |
| Threshold (mV) | -38.7 ± 2.3 | -39.8 ± 1.5 | -32.7 ± 5.1 |
| AP Peak (mV) | 53.8 ± 4.4 | 55.3 ± 3.7 | 65.6 ± 7.5 |
| Max. Rising slope (V/s) | 151.5 ± 18.0 | 194.5 ± 17.4 | 191.2 ± 44.6 |
| AP half-width (ms) | 1.3 ± 0.1 | 0.7 ± 0.1****** | 1.6 ± 0.2 |
| Max. repolarizing slope (V/s) | -54.4 ± 5.5 | -101.9 ± 17.2****** | -51.4 ± 6.8 |
| AHP (mV) | -11.6 ± 2.0 | -14.5 ± 2.2 | -13.3 ± 3.2 |
| Current threshold (pA) (5ms stimulus) | 653.7 ± 108.1  (n = 10) | 487.1 ± 51.4  (n = 7) | 1063 ± 116.4*****  (n = 4) |
| All data are presented as means ± s.e.m. *****p < 0.01; ******p < 0.001. Comparisons made vs. GFP using one-way ANOVA with by Bonferroni’s multiple comparisons test. | | | |
